# The Lysine Specific Demethylase-1 Negatively Regulates the *COL9A1* Gene in Human Articular Chondrocytes

**DOI:** 10.3390/ijms21176322

**Published:** 2020-08-31

**Authors:** Anne-Laure Durand, Alexandre Dufour, Elisabeth Aubert-Foucher, Christine Oger-Desfeux, Marielle Pasdeloup, Sebastien Lustig, Elvire Servien, Gualter Vaz, Emeline Perrier-Groult, Frederic Mallein-Gerin, Jerome E. Lafont

**Affiliations:** 1 CNRS UMR 5305 Laboratory of Tissue Biology and Therapeutic Engineering, Université Claude Bernard Lyon1, Univ Lyon, 69367 Lyon, France; anne-laure.durand@hotmail.fr (A.-L.D.); DUFOURA@tcd.ie (A.D.); elisabeth.aubert-foucher@ibcp.fr (E.A.-F.); marielle.pasdeloup@ibcp.fr (M.P.); emeline.groult@ibcp.fr (E.P.-G.); f.mallein-gerin@ibcp.fr (F.M-G.); 2 PRABI-AMSB, Batiment Mendel, Campus de la Doua, Université Claude Bernard Lyon1, University Lyon, 69622 Villeurbanne CEDEX, France; christine.oger@univ-lyon1.fr; 3 FIFA Medical Center of Excellence Orthopaedic Surgery and Sports Medicine Department, Croix-Rousse Hospital, Hospices Civils de Lyon, 103 grande rue de la Croix-Rousse 69317 Lyon CEDEX 04, France and IFSTTAR, LBMC UMR_T9406 Univ Lyon, Claude Bernard Lyon 1 University, 69317 Lyon, France; sebastien.lustig@gmail.com; 4 FIFA Medical Center of Excellence, Orthopaedic Surgery and Sports Medicine Department, Croix-Rousse Hospital, Hospices Civils de Lyon, 103 grande rue de la Croix-Rousse 69317 Lyon CEDEX 04, France; LIBM-EA 7424, Interuniversity Laboratory of Biology of Mobility, Claude Bernard Lyon 1 University, 69317 Lyon, France; elvire.servien@chu-lyon.fr; 5 Orthopaedic Surgery Department, CMCR les Massues, Croix rouge française, 92 rue Edmond Locard, 69005 Lyon, France; gualter.vaz@lyon.unicancer.fr

**Keywords:** articular chondrocytes, type IX collagen, lysine demethylase, osteoarthritis

## Abstract

Osteoarthritis (OA) is a degenerative disease of the joints which is associated with an impaired production of the cartilage matrix by the chondrocytes. Here, we investigated the role of Lysine-Specific Demethylase-1 (LSD1), a chromatin remodeling enzyme whose role in articular chondrocytes was previously associated with a catabolic activity and which is potentially involved during OA. Following a loss of function strategy and RNA sequencing analysis, we detail the genes which are targeted by LSD1 in human articular chondrocytes and identify *COL9A1*, a gene encoding the α1 chain of the cartilage-specific type IX collagen, as negatively regulated by LSD1. We show that LSD1 interacts with the transcription factor SOX9 and is recruited to the promoter of *COL9A1*. Interestingly, we observe that OA cartilage displays stronger LSD1 immunostaining compared with normal, and we demonstrate that the depletion of *LSD1* in OA chondrocytes prevents the decrease in *COL9A1* following Il-1β treatment. These results suggest LSD1 is a new regulator of the anabolic activity of articular chondrocytes potentially destabilizing the cartilage matrix, since it negatively regulates *COL9A1*, a gene encoding a crucial anchoring collagen molecule. This newly identified role played by LSD1 may thus participate in the alteration of the cartilage matrix during OA.

## 1. Introduction

Osteoarthritis (OA) is the most common joint disease and the major cause of impaired mobility in the aging population. One of the pathological features of the disease is the progressive and inevitable degeneration of articular cartilage, due to its poor regenerative capacity. The early onset of OA is not well understood, but the histological observation of the articular cartilage shows important changes in the extracellular matrix organization such as collagen degradation [1], and also cellular abnormalities such as proliferation or hypertrophy of the chondrocytes [2]. The extracellular matrix of articular cartilage is made of networks of proteoglycans and collagen fibrils including type II, IX, and XI collagen molecules [3]. This cartilage matrix is mainly produced by the resident chondrocytes, which are key players to maintain the mechanical properties of the articular cartilage and the overall joint integrity.

Among the cartilage-specific collagens, type IX collagen belongs to the fibril-associated collagen with interrupted triple helixes (FACIT), which greatly contribute to extracellular matrix organization by serving as molecular bridges. Indeed, type IX collagen molecules, through their triple helical domain, are linked to the surface of collagen fibrils [4]. More precisely, type IX collagen forms a heterotrimer with the α1, α2, and α3 chains and the α1 chain, encoded by the *COL9A1* gene, carries a globular domain responsible for the anchorage of the collagen fibrils to other non-collagenic molecules of the cartilage matrix, such as COMP [5,6]. Thus, type IX collagen is essential for normal development, notably protecting against premature vascularization in femoral heads and it was proposed as a regulating factor of angiogenesis during ossification of the femoral heads [7]. It is also important for the maintenance of several skeleton elements such as vertebral discs and cartilage as illustrated by the invalidation of the *Col9a1* gene in mice leads to important skeletal defects characterized by short stature and an OA-like phenotype [8,9,10,11].

The cartilage-specific genes are regulated by Sox9 (SRY-related protein 9). This transcription factor plays a critical role in cartilage formation because it induces the chondrocyte lineage at early steps of development [12]. Other studies have also shown that SOX9 expression is down-regulated in diseased cartilage such as OA [13,14]. Although this factor seems crucial in regulating several chondrocyte-specific genes by directly promoting the transcription of main matrix genes such as *COL2A1*, *COL9A1*, *COL11A2*, and *ACAN* [15,16,17,18], it regulates the transcription in close association with other SOX family proteins (L-SOX5 and SOX6) and probably through interactions with other regulators to be identified [19]. Among these, epigenetic factors have recently emerged as potential important players, notably as co-regulators of SOX9 [20]. Histone lysine methylation has thus emerged as a key player in many diseases and aging processes such as cancer [21] but very few data have been shown in degenerative diseases of cartilage such as OA. Epigenetic marks are catalyzed by chromatin-modifying enzymes in a dynamic and reversible manner. Chromatin-modifying enzymes are recruited to specific genomic regions by their interaction with transcription factors [22]. Generally, di- and tri-methylation of lysine 4 on histone 3 (denoted as H3K4), H3K36, and H3K79 correlate with the active state, while di- and tri-methylation of H3K9 and H3K27 correlate with the silent state [23]. However, little is known about the contribution of these enzymes to gene regulation in chondrocytes and to cartilage homeostasis.

In this study, we focused on the role of the Lysine-Specific Demethylase 1 (LSD1) [24] toward the regulation of the transcription of collagens in the articular chondrocytes. Interestingly, a previous study has shown that LSD1 is recruited on the promoter of the gene encoding the microsomal PGE synthase-1 (mPGES-1, protein involved in inflammation), and activates its transcription by demethylating H3K9 [25]. These observations demonstrate the contributive role of LSD1 in OA progression by stimulating the catabolic pathway in chondrocytes. The present report sought to investigate if LSD1 also controls the anabolic pathway.

We show that the histone demethylase LSD1 is recruited on the *COL9A1* gene promoter in its main regulatory region, negatively regulating the *COL9A1* transcription in human chondrocytes. Moreover, we provide evidence that the inhibition of LSD1 can prevent the negative regulation of *COL9A1* in IL-1ß-treated chondrocytes.

## 2. Results

### 2.1. LSD1 Labeling in OA Cartilage

**Figure 1:**
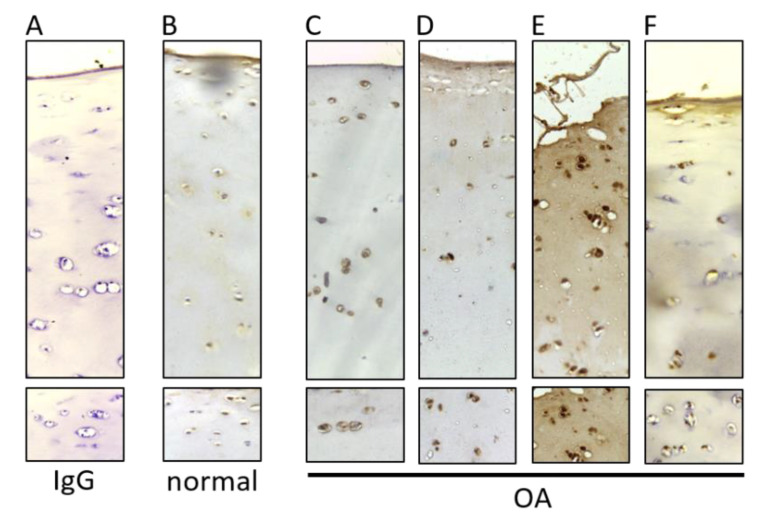
Protein detection of LSD1 in normal and osteoarthritis (OA) cartilage. Tissue sections of human cartilage obtained from non-diseased cartilage (**B**), or OA cartilage from several donors were immunostained for LSD1 (**C**–**F**). IgG was used as a control where the primary antibody was omitted (**A**) or not (**B**) and using OA cartilage.

To investigate whether LSD1 could be involved in OA, immunohistochemistry was performed on human articular sections of cartilage obtained from osteoarthritic and normal knee joints. While the presence of LSD1 was detected in the normal cartilage (Figure 1A,B), its presence seems more elevated in OA samples (Figure 1C–F), where a strong nuclear staining is observed. These histological observations were confirmed at the mRNA level for a limited number of patients (Figure S1) and seem to indicate that expression of LSD1 is correlated with OA.

### 2.2. LSD1 Depletion Leads to the Up-Regulation of the Expression of the Cartilage-Specific Gene COL9A1 in HACs

**Figure 2:**
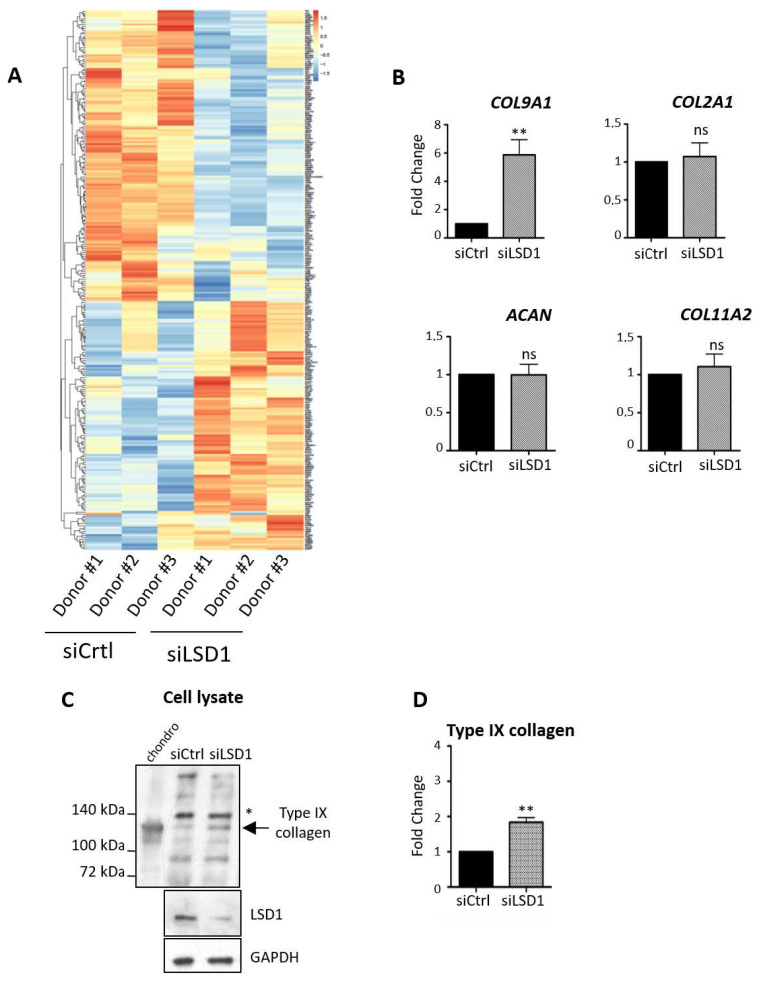
LSD1 down-regulates the expression of *COL9A1*. (**A**) Heatmap of the top 280 regulated genes following LSD1 depletion in articular chondrocytes obtained from three different donors. (**B**) Real-time PCR of a set of collagen encoding genes from at least 3 other independent experiments. Results are presented as relative mRNA levels of *COL9A1*, *COL2A1*, *COL11A2* and *AGGRECAN* (*ACAN*) normalized to *RPLP0* (Student’s *t*-test * *p* < 0.05, ** *p* < 0.01). (**C**) Protein expression of type IX collagen was analyzed by Western blot in cell lysates from human chondrocytes cultured after LSD1 knock-down. Mouse costal chondrocytes (chondro) were used as a positive control (*n* = 3). (**D**) Corresponding quantification was performed using ImageJ software (Student’s *t*-test ** *p* < 0.01).

In order to identify the genes that are regulated by LSD1, we performed an RNA sequencing experiment in *LSD1*-depleted primary chondrocytes. Differential expression analysis revealed that 280 genes were differentially expressed between LSD1-depleted and control chondrocytes, among which 128 were up-regulated and 152 were down-regulated upon siRNA treatment (Figure 2A). We performed a supervised analysis by looking at the SOX9 target genes previously described in the literature. Among the collagen encoding genes, only one, *COL9A1*, was importantly and significantly up-regulated in LSD1-depleted chondrocytes (Supplemental dataset1), indicating that LSD1 exerts a negative regulation on *COL9A1* in human chondrocytes. Real-time PCR experiments were performed on additional donors and confirmed the inhibition of the transcription of *COL9A1* mRNA (4- to 6-fold), whereas other collagens (such as *COL2A1* or *COL11A2*) or *AGN* (which is a well-characterized SOX-9-dependent gene) were not significantly modified (Figure 2B), showing that LSD1 specifically inhibits the transcription of *COL9A1*. The up-regulation of type IX collagen protein synthesis was also confirmed by Western blot in cell lysates (Figure 2C) of LSD1-depleted chondrocytes. Quantified Western blot shows the up-regulation reaches up to an almost 3-fold increase in cell lysates (Figure 2D).

### 2.3. LSD1 Does not Affect the Level of Expression of SOX9 or Its Stability but Associates with SOX9 Protein

Since LSD1 is known to regulate gene expression through interaction with several transcription factors [26], we studied its role toward SOX9, a key transcription factor that controls the synthesis of cartilage matrix proteins.

**Figure 3:**
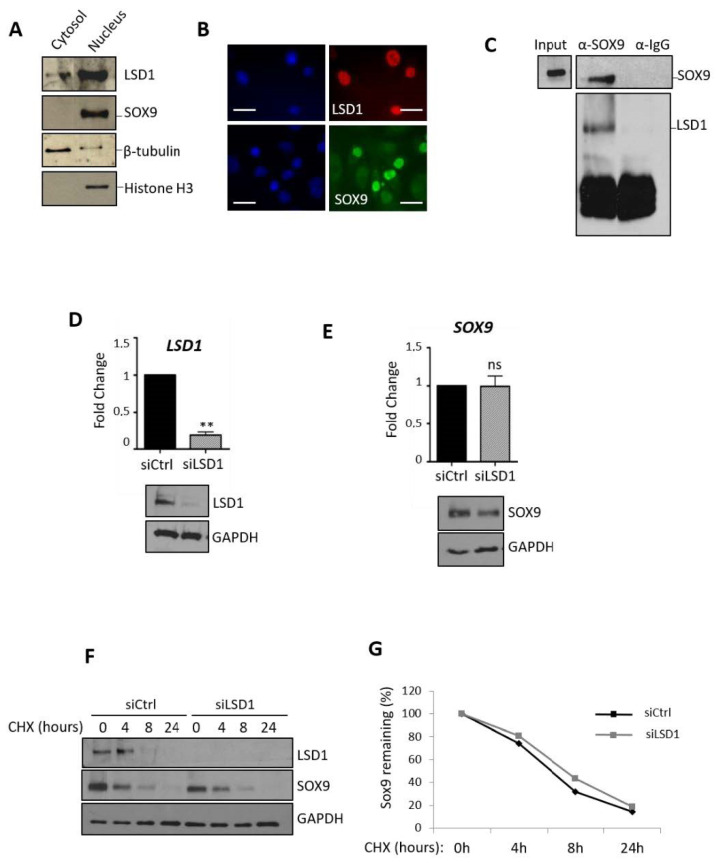
LSD1 interacts with SOX9 in human chondrocytes. (**A**) Cytosolic and nuclear fractions were analyzed by Western blot and tested for the expression of SOX9 and LSD1. β-tubulin and Histone H3 were used as cytosolic and nuclear markers, respectively. (**B**) Localization of LSD1 and SOX9 proteins was assessed by immunofluorescence analysis (scale bar: 10µm). Nuclei were counterstained with Hoechst (left panels). (**C**) Immunoprecipitation of SOX9 was realized using an anti-SOX9 antibody, then anti-LSD1 was probed to test if it co-immunoprecipitates (*n* = 3). (**D**,**E**) Knock-down of LSD1 was realized following transfection of human chondrocytes with siRNA. Real-time PCR was performed from at least 5 independent experiments. Results are presented as relative mRNA levels of *LSD1* and *SOX9* normalized to *RPLP0*, after LSD1 knock-down in human chondrocytes (Student’s *t*-test ** *p* < 0.01, ns: non significant). Western blot was performed to detect LSD1 or SOX9 protein expression after siRNA transfection. (**F**) Protein synthesis was inhibited with cycloheximide (CHX) incubation and a time course from 0 to 24 h was performed prior to the analysis of SOX9 protein stability by Western blot (*n* = 3). (**G**) Corresponding quantification was performed using ImageJ software.

As a first step, we analyzed the subcellular distribution of LSD1 and SOX9 in human articular chondrocytes (HAC). Western blots of nuclear and cytosolic fractions of human primary chondrocytes indicate that both proteins, SOX9 and LSD1, are mainly present in the nuclear fraction (Figure 3A). A similar localization is detected by immunofluorescence (Figure 3B). Since LSD1 was previously shown to be a transcriptional co-regulator, we tested the eventual interaction between SOX9 and LSD1. As assessed in Figure 3C, the endogenous SOX9 protein was immunoprecipitated. Interestingly, immunoblot against LSD1 shows that the demethylase is also present in the immunoprecipitated fraction, thus demonstrating that LSD1 interacts with SOX9 in chondrocytes isolated from human articular cartilage. Next, the expression of *LSD1* and *SOX9* transcripts was examined according to the status of the chondrocyte phenotype. Whilst the mRNA level of *SOX9* drops with the dedifferentiation of chondrocytes provoked by successive passages, we observed that the level of *LSD1* transcripts remains stable (Figure S2). Similarly, no change of *LSD1* mRNA level was observed when the redifferentiation is enhanced such as during hypoxic or 3D culture (Figure S2). Thus, LSD1 interacts with SOX9, although its expression does not correlate with the status of the chondrocyte phenotype.

To further study the role of LSD1 as a new partner of SOX9 protein, we then asked if LSD1 could regulate SOX9 expression through a loss of function approach. The depletion of LSD1 was thus realized following the transfection of primary chondrocytes with LSD1-targeting siRNA. qPCR experiments show that nearly 80% knock-down was obtained using siRNAs targeting *LSD1* mRNA (Figure 3D). This leads to the almost complete absence of LSD1 protein. We then assessed the consequence of LSD1 depletion on the synthesis of *SOX9* mRNA and protein. Results in Figure 3E show that the mRNA level is unchanged whereas the protein level is modestly decreased when looking at the protein level after transfection with the siRNA (Figure 3E). These data show that the depletion of LSD1 does not affect the steady-state level of SOX9 at the mRNA and protein levels. Since LSD1 can also affect the protein stability of non-histone proteins, we next asked whether LSD1 could influence SOX9 protein stability. Following a similar strategy of siRNA-driven LSD1 depletion and treatment with cycloheximide (CHX), SOX9 protein was analyzed by Western blot (Figure 3F). We observed that the decay of SOX9 protein is the same whatever the level of LSD1 (Figure 3G), suggesting that the half-life of SOX9 is unchanged in the absence of LSD1. These results indicate that LSD1 interacts with SOX9, however it does not affect the level or the stability of SOX9.

### 2.4. LSD1 Is Recruited onto the SOX9- Binding Region of the COL9A1 Promoter

**Figure 4:**
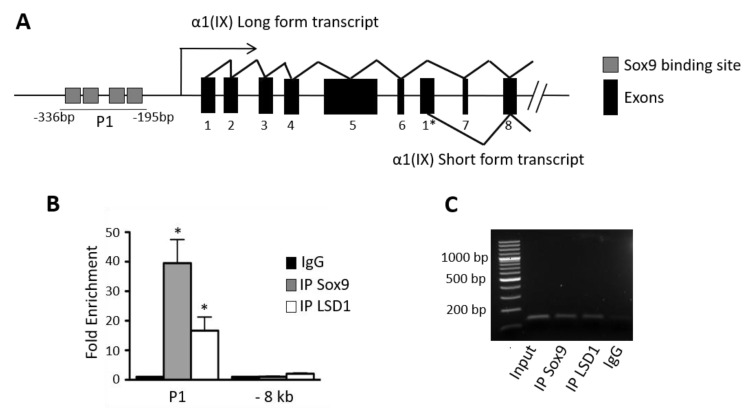
LSD1 is recruited onto the *COL9A1* promoter region. (**A**) Schematic representation of the *COL9A1* promoter which contains four SOX9 binding sites arranged in two pairs. Primers used for PCR are indicated (denoted P1 and -8kb). (**B**) Immunoprecipitation of chromatin was done using anti-SOX9 or anti-LSD1 antibody from human articular chondrocytes. Then, real-time PCR was performed using specific primers P1 of the *COL9A1* promoter regions or 8kb upstream (*n* = 3, Student’s *t*-test * *p* < 0.05). (**C**) Agarose gel shows the corresponding PCR products using the P1 primers.

In order to describe the mechanisms of this regulation, we next examined whether LSD1 was recruited onto the promoter of *COL9A1.* We focused on the promoter region that controls the long form transcript of *COL9A1*, which is the cartilage-specific form. This region is 330 bp upstream the transcription starting site (TSS) and contains functional SOX9 binding sites as previously described [27] (Figure 4A). Following chromatin immunoprecipitation with a SOX9 antibody, and using primers encompassing the SOX9 binding site region (P1), an important PCR product was amplified, demonstrating as expected, the endogenous SOX9 protein is recruited on the *COL9A1* gene promoter (Figure 4B and C). Performing an immunoprecipitation of the chromatin with an anti-LSD1 antibody, an important PCR amplification of the same region (P1) was also obtained, thus showing a physical occupancy of LSD1 protein on the promoter of *COL9A1* (Figure 4B,C). As a negative control, we did not detect any enrichment of either LSD1 or SOX9 at 8kb upstream of the TSS. Altogether, these data obtained in human OA chondrocytes show the endogenous recruitment of LSD1 occurs in the region of the *COL9A1* promoter, which contains the SOX9 binding sites and where SOX9 is also recruited. It is the first evidence that LSD1 down-regulates *COL9A1*, a collagen encoding gene which is cartilage-specific.

### 2.5. The Down-Regulation of the COL9A1 Gene Expression by the Inflammatory Cytokine IL1-β Is Blocked When LSD1 Is Depleted

**Figure 5:**
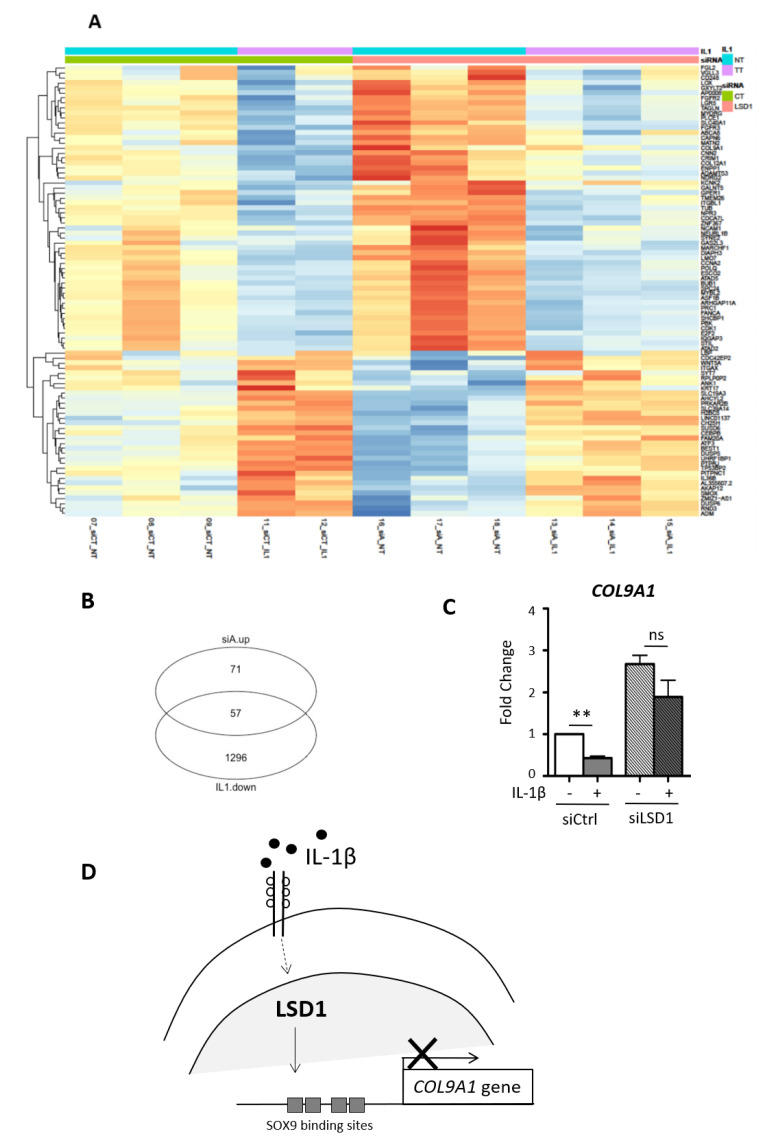
*COL9A1* mRNA levels in LSD1-depleted chondrocytes in response to the proinflammatory cytokine IL-1β. Knock-down of LSD1 was realized following human chondrocyte transfection with siRNA and then treated with IL-1β for 24 h. (**A**) Heatmap of the genes oppositely regulated by LSD1 and IL-1β in articular chondrocytes (NT: non-treated, TT: treated with IL-1β; CT: transfected with siControl, LSD1: transfected with siRNA against LSD1). (**B**) Diagram indicates 57 genes are simultaneously down-regulated by IL-1β and up-regulated following LSD1 depletion. (**C**) Real-time PCR was performed from at least 3 independent experiments of additional donors. Results are presented as relative mRNA levels of *COL9A1* normalized to *RPLP0 (n* = 4, Student’s *t*-test * *p* < 0.05 ** *p* < 0.01). (**D**) Schematic representation showing the down-regulation of *COL9A1* by IL-1β is mediated by LSD1.

Due to the potential role of LSD1 during OA, we then evaluated if the inhibition of LSD1 can prevent the effect of the inflammatory cytokine IL-1β in LSD1-depleted chondrocytes. RNA sequencing revealed that IL-1ß stimulation differentially regulated the expression of 2757 genes (1405 were up-regulated, 1352 down-regulated, Figure S3). As expected, our supervised analysis indicates that IL-1β stimulates several catabolic genes such as *mPGES1* or the aggrecanase *ADAMTS-4*, and inhibits the cartilage matrix encoding genes such as *COL2A1* and *COL9A1* (Supplemental dataset2). More interestingly, further analysis of the RNA sequencing shows the genes that were oppositely regulated by IL-1β and by LSD1 (Figure 5A), and reveals that 57 genes were simultaneously down-regulated by IL-1β and up-regulated by LSD1 depletion (Figure 5B). Interestingly, we identified *COL9A1* as one of these and could confirm by real-time PCR analysis (performed on additional donors) that the negative regulation of IL-1β on *COL9A1* is no longer detected when LSD1 is inhibited (Figure 5C). These results thus show that the demethylase LSD1, which is abundantly detected in OA cartilage, participates in the decrease in *COL9A1* in the articular chondrocytes stimulated by the proinflammatory cytokine IL-1β. 

## 3. Discussion

Increasing evidence indicates that the epigenetic factors, such as the lysine-specific histone demethylase 1, may be involved in OA through their control of gene expression [28]. In this study, we describe a new role for LSD1 in the homeostasis of human articular chondrocytes since we demonstrate that this histone demethylase is responsible for the down-regulation of *COL9A1* gene.

Through the RNA sequencing analysis, we established a list of the genes regulated by LSD1 in human chondrocytes and found that LSD1 depletion affects *COL9A1* expression and no other SOX9-dependent collagens (supplemental dataset1 and Figure 3). These results are confirmed by qPCR and we observed similar results with two different siRNA. The epigenetic regulations rely on the specific marks of chromatin harbored within each promoter [29], which may explain the specific effect of LSD1 toward *COL9A1* (no other cartilage-specific collagens are modified). Indeed, to date, *COL9A1* is the only cartilage-specific collagen gene whose expression is regulated by epigenetics such as DNA methylation [30]. However, in our RNAseq analysis, other genes previously associated with OA such as *SOX4*, *HIF-2α*, or *VEGF*, are down-regulated following LSD1 inhibition, suggesting that this enzyme exerts a control of the cartilage integrity beyond the regulation of *COL9A1*. The in vivo investigation of its role toward osteoarthritis progression would thus be of great interest.

We also report the first evidence of an interaction between the demethylase LSD1 and the transcription factor SOX9 in human cells. It was previously shown that SOX9 interacts with the co-regulator ARID5B to recruit the demethylase PHF2 [20], however it is the first demonstration of an interaction between SOX9 and a demethylase.

We raised the possibility that this association could regulate the expression or the stability of SOX9 protein. However, we did not observe any effect of LSD1 depletion on *SOX9* expression in human chondrocytes, contrary to Zhang et al., who showed that LSD1 represses *Sox9* expression in mouse chondrocytes [31] probably due to different mechanisms between species. Since we also found that LSD1 does not affect SOX9 protein stability, we analyzed the effect of LSD1 on SOX9 activity by looking at its known target genes. The depletion of LSD1 increases the *COL9A1* expression, indicating that LSD1 exerts a negative regulation on *COL9A1* transcription, in accordance with previous data describing LSD1 as a transcriptional repressor [26]. Thus, LSD1 as a new partner of the SOX9 machinery would represent a new transcriptional co-repressor in chondrocytes. 

As an explanatory mechanism of how LSD1 down-regulates *COL9A1*, we provide evidence of endogenous recruitment of LSD1 onto the *COL9A1* promoter. This recruitment is located where functional SOX9 binding sites are also located (Figure 4B), which suggests that LSD1 directly modulates the methylation of histone proteins nearby important cartilage-specific regulatory elements. Since LSD1 is unable to directly bind to DNA [32], it can be concluded that the interaction between LSD1 and SOX9 allows LSD1 to be recruited on the *COL9A1* promoter, which is a mechanism of action already described for LSD1 [33,34], notably through the demethylation of H3K4 [35]. Another possible role for LSD1 would come from altering the transcriptional activity of SOX9 itself, since it has been demonstrated that LSD1 can modulate the activity of non-histone proteins through their demethylation [36]. Whether the methylation of SOX9 is modified by LSD1 was not investigated in this study, but the methylation of SOX9 protein, whose effect is still unknown, has been previously shown [37]. Interestingly, the methylation sites of SOX9 are located nearby the phosphorylation sites, which regulate the activity of SOX9 [38]. Only the methylations at arginine residues have been identified so far, and a recent study demonstrated that several histone lysine demethylases can catalyze arginine demethylation [39]. The interaction of SOX9 with a demethylase reinforces the existence of such a post-translational modification, and further studies have to be performed in order to determine if SOX9 is a substrate of LSD1.

We observe a higher protein staining for LSD1 in OA cartilage samples compared with normal cartilage. These results are in agreement with previous observations obtained by El Mansouri et al. [25] and suggest that the level of LSD1 may increase in the cartilage during OA and could be responsible for the altered expression of type IX collagen. Indeed, it is well established that type IX collagen synthesis decreases in OA cartilage [30,31,32,33,34,35,36,37,38,39,40], which has important consequences on cartilage homeostasis: Col IX-deficient mice have profound consequences on matrix composition and anchorage in the cartilage of new-born animals, which may affect the mechanical properties of that tissue and may predispose for cartilaginous degeneration in older animals [41]. More recently, it has also been shown that the absence of Col-IX leads to premature vascularization [7]. Since inflammation is an important driver of OA, we investigated if LSD1 depletion could prevent some inflammatory effects of IL-1β. Interestingly, through our RNA sequencing, we observe that from the 57 genes that were simultaneously down-regulated by IL-1β and oppositely up-regulated by LSD1 depletion, the *COL9A1* gene was present (Figure 5A and B). Conversely, we also observe 33 genes whose expression is up-regulated by IL-1β and down-regulated by LSD1 depletion. Interestingly, catabolic genes previously involved in OA such as *WNT5A* [42] or *ADAMTS-3* [43], or inflammatory genes such as *IL-36β* [44] were among these, suggesting LSD1 exerts broader effects not only decreasing the expression of key matrix genes but also increasing catabolic genes. These data reinforce the role of LSD1 as a bipotent promoter of OA since El Mansouri et al. [25] previously showed that LSD1 mediates catabolic signals of IL-1β through enhancing the expression of the prostaglandin synthase enzyme *mPGES-1*.

This is, to our knowledge, the first report of a role of LSD1 in the control of the ability of human chondrocytes to synthesize one cartilage-specific collagen, the *COL9A1* encoding gene; in addition to its role in enhancing the expression of inflammatory factors such as mPGES1, LSD1 represses type IX collagen synthesis. Thus, it is tempting to hypothesize that LSD1, following an enhanced level in OA, could have a dual role in the alteration of cartilage matrix, one against anabolism and the other contributing to catabolism (Figure 5D). With this view, prevention of OA through the inhibition of LSD1 would be an interesting strategy to consider.

## 4. Materials and Methods

### 4.1. Cartilage Samples

Human osteoarthritic articular cartilage was obtained from patients undergoing knee arthroplasty (age range 61–90) and was used for the histology and primary culture of chondrocytes (as detailed below). Normal articular cartilage was obtained from the femoral condyle of patients with no history of joint disease (age range 32–68) and was used only for histology. The study was performed in full accordance with local ethics committee guidelines and all the cartilage samples were collected after written and informed consent of the donors according to local legislation. All the experimental protocols were approved by the French ministry of higher education and research (ethics committee for research with human samples CODECOH: DC 2014–2325).

### 4.2. Chondrocyte Isolation and Primary Monolayer Culture

The cartilage from OA joints was sliced into small pieces (2 mm^3^) and digested in culture medium consisting of DMEM/F-12 (Invitrogen, MA, USA) with 0.5 mg/mL of collagenase A (Roche Applied Science, Weilheim-Schongau, Bavaria, German) overnight at 37 °C. The cell suspension was filtered, and isolated chondrocytes were then seeded in DMEM/F-12 supplemented with 10% FBS and antibiotics at 37 °C in 5% CO_2_. At this stage, human articular chondrocytes (HAC) were designated primary and were cultured for no more than one passage (P1) to avoid complete chondrocyte dedifferentiation. For treatments, cycloheximide (Sigma-Aldrich St. Louis, MO, USA) was used at 100 μg/mL and IL-1β (PeproTech, Rocky Hill, NJ, USA) at 50 pg/mL for 24 h. 

### 4.3. Western Blot

Human articular chondrocytes were rinsed with phosphate buffered saline (PBS), then lysed and boiled in Laëmmli buffer 1X (Bio-Rad). Equivalent amounts of proteins were separated on SDS-PAGE (4–12% gradient gels Bio-Rad). Proteins were then transferred to an Immobilon PVDF membrane (Millipore) and probed with specific primary antibodies against SOX9 (AB5535, Millipore 1/1000), type IX collagen (MAB3304 Millipore, 1/3000), LSD1 (208–13-1-AP, Proteintech, 1/750), Tubulin (T8535, Sigma, 1/2000) or GAPDH (sc-81545, Santa Cruz, 1/2000) overnight at 4 °C after saturation. Then, alkaline phosphatase-conjugated IgG antibodies (anti-mouse or anti-rabbit, Cell signaling Danvers, MA, USA) were incubated one hour at room temperature. Proteins were visualized on X-rays films (Sigma-Aldrich, St. Louis, MO, USA) using an Immun-star AP chemiluminescent substrate (Bio-Rad Hercules, CA, USA).

### 4.4. Immunofluorescence

Human articular chondrocytes were seeded on glass coverslips in six-well plates at 25 × 10^4^ cells/well. After 24 h, cells were fixed with 4% paraformaldehyde for 30 min at 4 °C, permeabilized with 0.1% TritonX-100 for 20 min at room temperature, and incubated with blocking buffer containing 1% bovine serum albumin (BSA) for 30 min. The SOX9 and LSD1 proteins were labeled overnight at 4 °C with the primary antibodies against SOX9 (AB5535, Millipore, Billerica, MA, USA 1/100) and LSD1 (05–939, Millipore, 1/50). The conjugated secondary antibody (Invitrogen) was labeled for another one hour at room temperature.

### 4.5. Transient Transfection

One day before transfection, human articular chondrocytes were seeded at 1 × 105 cells/well in six-well culture dishes. Cells were transfected with 30 nM of siRNA against LSD1 or control (Qiagen, Hilden, Germany) using Lipofectamine 2000 (Invitrogen) in serum-free OptiMEM. After 24 h, the culture medium was changed. 

### 4.6. Subcellular Fractionation

A total of 5 × 10^5^ human articular chondrocytes were seeded 24 h before cells were washed and scraped in buffer A (10 mM Hepes pH 7.9, 1.5mM MgCl_2_, 10 mM KCl, 10 μg/mL leupeptin, 10 μg/mL aprotinin, 1 mM Na_3_VO_4_, 1 mM PMSF) before the pellet was again resuspended in 100 μL of buffer A. The cells were incubated on ice for 10 min. Following centrifugation, the cytosolic fraction (supernatant) was collected. Subsequently, the pellet was resuspended on ice for 20 min in 50 μL of buffer C (20 mM Hepes pH 7.9, 1.5 mM MgCl_2_, 420 mM NaCl, 0.2 mM EDTA, 25% glycerol, 10 mM KCl, 10 μg/mL leupeptin, 10 μg/mL aprotinin, 1 mM NaVO4, 1mM PMSF). After centrifugation, the supernatant fraction representing the nuclear extract was collected.

### 4.7. Co-Immunoprecipitation 

A total of 1.5 × 10^6^ human articular chondrocytes were seeded 24 h before cells were harvested and lysed with a NP-40 lysis buffer (50 mM Hepes pH8, 150 mM NaCl, 5 mM EDTA, 1% NP-40) supplemented with protease and phosphatase inhibitors (10 μg/mL leupeptin, 10 μg/mL aprotinin, 1 mM NaVO4, 1mM PMSF) for 15 min on ice. An amount of 500 μg of total proteins was incubated with 3 μg of anti-SOX9 antibody (Millipore) for 30 min at 4 °C and conjugated to Protein A-Agarose (Roche). After washing, the precipitated proteins were eluted and analyzed by Western blot.

### 4.8. Real-Time PCR Analysis

Total RNA from cultured human articular chondrocytes was isolated using the NucleoSpin^®^ RNA kit (Macherey-Nagel, Bethlehem, PA, USA) according to the manufacturer’s instructions. An amount of 150ng was reverse-transcribed into cDNA by the PrimeScript™ RT reagent Kit (TaKaRa, Mountain View, CA, USA) in a total volume of 20 µL. Diluted cDNA was analyzed by real-time quantitative PCR using FastStart Universal SYBR Green Master (Roche) in a Rotor-Gene Q system (Qiagen). The specific primers used for RT-qPCR analysis are shown in Table 1. Results are expressed as relative values normalized with the reference gene ribosomal protein (*RPL30*) and quantified by the 2^−ΔΔCt^ method.

### 4.9. RNA Sequencing and Bioinformatic Analysis

Human articular chondrocytes from three independent primary cultures (three donors) were subjected to transient transfection of siRNA against LSD1 or control (as described above) and either treated or not with IL1-ß. RNA from these 12 samples was extracted using the NucleoSpin^®^ RNA kit (Macherey-Nagel, Bethlehem, PA, USA), qualified on a fragment analyzer (Proteigene) and quantified by fluorescence with Quantus apparatus (Promega, Fitchburg, WI, USA). Libraries preparations were performed on all but one poor sample by using 0.5 µg total RNA and the NextFlex rapid Directional mRNA-seq (Bio Scientific, Phoenix, AZ, USA) according to the manufacturer’s instructions, and RNA-Seq was performed by the core facility ProfileXpert (Lyon, France). Libraries were sequenced using the NextSeq 500 sequencing platform (Illumina) by generating 75bp single-end reads. A minimum of 20 million reads were obtained per sample. FastQC [45] and MultiQC [46] were used to check the quality of sequencing data. No trimming of sequences was needed. Reads were aligned to the human genome using HISAT2.1.0 (index grch38-tran) and assigned and counted on annotated Ensembl genes (release 84) using featureCounts v1.6.0 [47,48]. Normalization, multivariate exploration (PCA) for the quality check of count data, and differential analyses were conducted with DESeq2 v1.24.0 [49]. The model applied to fit data included a cell line effect, and principal and interaction effects for Lsd1 depletion and IL-1β treatment (design *n* = ~ cell + siA + IL1 + siA:IL1). To estimate the log fold change (LFC) of gene expression, the apeglm version of LFC shrinkage was applied. Genes with a false discovery rate (FDR) below 0.05 and a differential effect of at least 50 percent (|LFC| > log_2_(1.5)) were considered as significantly differentially expressed. The lists of regulated genes are presented in additional files 3–5. Heatmaps of variance-stabilized data (with vst function from DESeq2) were represented using the pheatmap package (colors are scaled by row).

### 4.10. Chromatin Immunoprecipitation (ChIP) Assay

Human articular chondrocytes were crosslinked in 1% formaldehyde for 15 min, washed with PBS, and resuspended in lysis buffer (1% SDS, 10 mM EDTA, 50 mM Tris-HCl, pH 8.1). Chromatin was sheared and extracts were diluted in the ChIP dilution buffer (0.01% SDS, 1.1% Triton X-100, 1.2 mM EDTA, 16.7 mM Tris-HCl, pH 8.1, 167 mM NaCl) and pre-cleared for one hour on Protein A-Agarose at 4 °C with rotation. After centrifugation (5000× *g* for 1 min), the supernatant was incubated with 3 μg of specific antibodies (SOX9, LSD1: Millipore) overnight at 4 °C with rotation followed by the addition of Protein A-Agarose (Roche) for another one hour. Beads were washed once with a low-salt buffer (0.1% SDS, 1% Triton X-100, 2 mM EDTA, 20 mM Tris-HCl, pH 8.1, 150 mM NaCl), and with a high-salt buffer (0.1% SDS, 1% Triton X-100, 2 mM EDTA, 20 mM Tris-HCl, pH 8.1, 500 mM NaCl), then twice with TE buffer (10 mM Tris-HCl, 1 mM EDTA, pH 8.0). Chromatin was collected in SDS/NaHC03 buffer, then reverse-crosslinked at 65 °C overnight followed by RNase A and proteinase K treatments. DNA was purified using a QIAquick PCR purification kit (Qiagen) and eluted with water. The precipitated DNA fragments were analyzed by RT-qPCR with primers designed to amplify specific regions of the *COL9A1* promoter (Table 1). Enrichments were calculated as fold changes relative to control conditions using the 2^−∆∆CT^ method.

### 4.11. Immunohistochemistry

Cartilage specimens from OA (OA joint) or healthy cartilage (healthy joint) were fixed in Alcohol-Formol-Acetic acid and embedded in paraffin. After deparaffinization, the sections were permeabilized with a pre-heated 0.3% triton X-100 and incubated with hyaluronidase (800 U/mL Sigma-aldrich) for 30 min at 37 °C. After several washes, the sections were immersed for 10 min to quench endogenous peroxidase activity (Vector Laboratories, Burlingame, CA, USA). Sections were blocked with 2.5% normal horse Serum for 1h and subsequently incubated with anti-LSD1 antibody overnight at 4 °C. The samples were then counterstained with hematoxylin and covered for observation with a DM 4000B microscope (Leica) coupled to a color camera (Digital Camera DXM1200, Nikon).

### 4.12. Statistical Analysis

Results of the real-time PCR are expressed as the mean ± SD from at least five independent experiments (or three independent experiments in the case of the ChIP assay), and statistical significance was assessed by Student’s *t*-test. *p* values less than 0.05 were considered statistically significant.

## Figures and Tables

**Table 1:** List of primers used for PCR experiments.

Application	Gene	Forward Primer	Reverse Primer
qPCR	*LSD1*	5′-TGAGAAAATCCACGCTGGCA -3′	5′-TCCTCCCTGTGCTCTAGGTC-3′
	*SOX9*	5′-ACGCCGAGCTCAGCAAGA-3′	5′-CACGAACGGCCGCTTCT-3′
	*COL2A1*	5′-TCCATGTTGCAGAAAACCTTCA-3′	5′-GGAAGAGTGGAGACTACTGGATTGAC-3′
	*COL9A1*	5′-ACGGTTTGCCTGGAGCTAT-3′	5′-ACCGTCTCGGCCATTTCT-3′
	*COL11A2*	5′-CCTGACCCACTGA GTATGTTCAT-3′	5′-TTGCAGGATCAGGGAAAGTGA-3′
	*ACAN*	5′-TCGAGGACAGCGAGGCC-3′	5′-TCGAGGGTGTAGCGTGTAGAGA-3′
	*RPL30*	5′-CCTAAGGCAGGAAGATGGGGTG-3′	5′-AGTCTGCTTGTACCCCAGGA-3′
ChIP	*COL9A1* (P1)	5′-CCTCCCAGTGGGCACATTTT-3′	5′-TCCAGGCAAGGTGCTATAGGAACA-3′
	−8 kb	5’-AGCTGTTTGACTGGTTCACCA-3’	5’-TACCTCGCAACAATCCAGCA-3’

## Supplementary Materials

Supplementary materials can be found at https://www.mdpi.com/1422-0067/21/17/6322/s1. Figure S1: Expression level of LSD1 in human articular chondrocytes from normal or OA cartilage. Figure S2: Expression level of LSD1 and SOX9 following dedifferentiation of human chondrocytes or their redifferentiation under hypoxic environment or 3D culture. Figure S3: Heatmap of the regulated genes following IL-1β treatment of articular chondrocytes obtained from two different donors. Supplementary dataset 1: List of the regulated genes following LSD1 depletion in articular chondrocytes (*n* = 3). Supplementary dataset 2: List of the regulated genes following IL-1β treatment of articular chondrocytes.
