# Supplementary figures and images for: The Lysine Specific Demethylase-1 Negatively Regulates the COL9A1 Gene in Human Articular Chondrocytes
Source: Int J Mol Sci. 2020 Aug 31;21(17):6322. doi: 10.3390/ijms21176322 (PMC7504057; doi:10.3390/ijms21176322)

**Fig. S1**

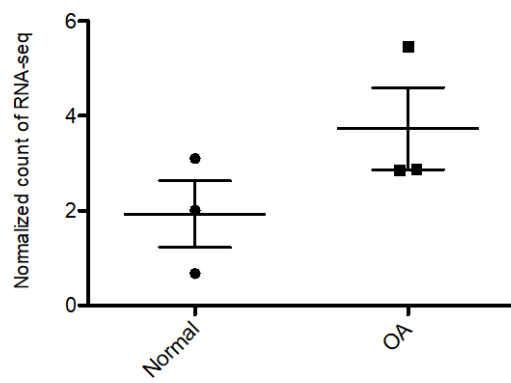

Fig. S2

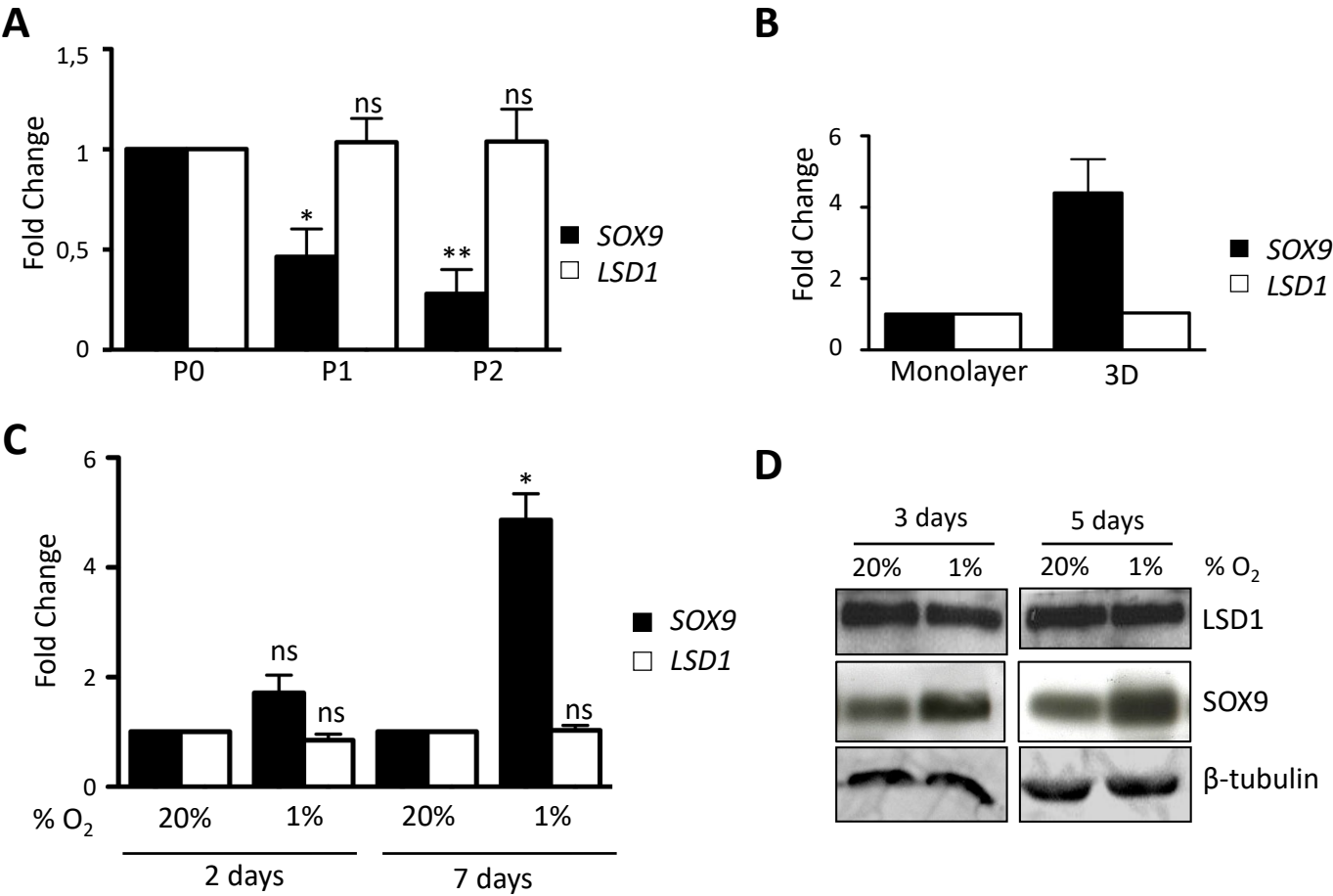

Fig. S3

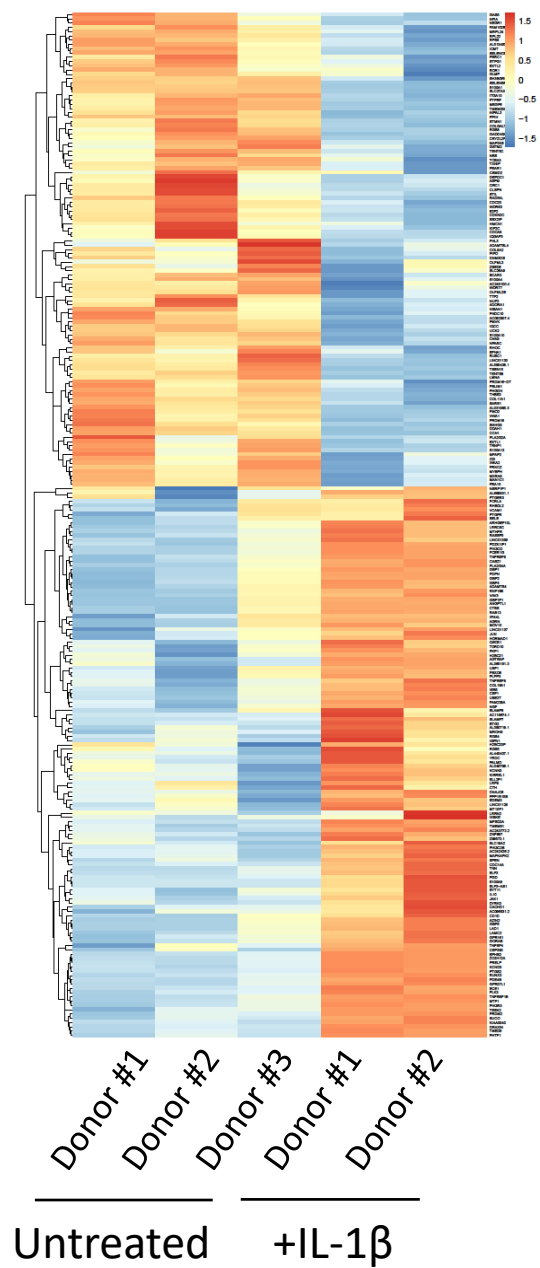

Supplement: Supplementary file 1 [file ijms-21-06322-s001.zip › figures S123.pdf]
